# De-identification of clinical notes with pseudo-labeling using regular expression rules and pre-trained BERT

**DOI:** 10.1186/s12911-025-02913-z

**Published:** 2025-02-17

**Authors:** Jiyong An, Jiyun Kim, Leonard Sunwoo, Hyunyoung Baek, Sooyoung Yoo, Seunggeun Lee

**Affiliations:** 1 Graduate School of Data Science, Seoul National University, Seoul, South Korea; 2 Department of Radiology, Seoul National University Bundang Hospital, Seongnam, South Korea; 3 Healthcare ICT Research Center, Office of eHealth Research and Businesses, Seoul National University Bundang Hospital, Seongnam, South Korea

**Keywords:** De-identification, Natural language processing, Clinical documentation and communications, Electronic health records and systems

## Abstract

**Background:**

De-identification of clinical notes is essential to utilize the rich information in unstructured text data in medical research. However, only limited work has been done in removing personal information from clinical notes in Korea.

**Methods:**

Our study utilized a comprehensive dataset stored in the Note table of the OMOP Common Data Model at Seoul National University Bundang Hospital. This dataset includes 11,181,617 radiology and 9,282,477 notes from various other departments (non-radiology reports). From this, 0.1% of the reports (11,182) were randomly selected for training and validation purposes. We used two de-identification strategies to improve performance with limited and few annotated data. First, a rule-based approach is used to construct regular expressions on the 1,112 notes annotated by domain experts. Second, by using the regular expressions as label-er, we applied a semi-supervised approach to fine-tune a pre-trained Korean BERT model with pseudo-labeled notes.

**Results:**

Validation was conducted using 342 radiology and 12 non-radiology notes labeled at the token level. Our rule-based approach achieved 97.2% precision, 93.7% recall, and 96.2% F1 score from the department of radiology notes. For machine learning approach, KoBERT-NER that is fine-tuned with 32,000 automatically pseudo-labeled notes achieved 96.5% precision, 97.6% recall, and 97.1% F1 score.

**Conclusion:**

By combining a rule-based approach and machine learning in a semi-supervised way, our results show that the performance of de-identification can be improved.

**Supplementary Information:**

The online version contains supplementary material available at 10.1186/s12911-025-02913-z.

## Background

Clinical notes contain detailed information regarding the medical history and current health status of patients. As Electronic Health Record (EHR) systems have been widely adopted both globally [1] and in Korea [2, 3], the number of narrative clinical notes in EHR has also increased. Although these notes are valuable resources for medical research, they cannot be openly shared, as they contain protected health information (PHI). Sharing clinical notes with PHI can have severe repercussions for patients, such as breaches of privacy and risks of identity theft. Furthermore, institutions responsible for the unauthorized sharing of these notes will face regulatory penalties. Such breaches can also result in a significant erosion of public trust in the institution’s capability to safeguard data. The de-identification of clinical notes is pivotal for the broader use of clinical data in research. For example, important clinical information required for patient diagnosis, assessment, and treatment planning, such as radiology reports and pathology reports, are recorded in unstructured or semi-structured text which limits data access and utilization for research purposes. Effective de-identification practices allow researchers to access vast datasets without compromising individual privacy.

De-identifying PHI in texts has been extensively studied. A conventional approach is to build hand-crafted rules, such as regular expressions and dictionary look-ups. For example, Medical Information Mart for Intensive Care (MIMIC) database de-identifies clinical notes using extensive dictionary look-ups and pattern-matching with regular expressions [4]. Using publicly available data, Philter-ucsf [5] software has a vast vocabulary and regular expression set to remove PHI. With the rapid development of machine learning methods, data-driven approaches have been developed for PHI detection, including support vector machines (SVM) [6], conditional random fields (CRF) [7], and deep learning [8]. After the release of BERT in 2018, BERT-based pre-trained language models, such as BioBERT [9] and ClinicalBERT [10] were developed for the clinical domain and used for PHI identification. Meaney et al. [11] (2022) confirmed that BERT-based models provide adequate solutions for the clinical-deidentification problem by evaluating the de-identification performance of various BERT-based models on clinical text data.

Recent studies on de-identification in English clinical narrative texts highlight significant advances and challenges. Yang et al. (2019) investigated deep learning-based de-identification methods in a cross-institute setting, identifying customization using local clinical text as a crucial factor for improving model performance. Hartman et al. (2020) assessed various machine learning systems for clinical note de-identification, emphasizing the effectiveness of fully customized systems. Johnson et al. (2020) developed and evaluated a de-identification approach using a bidirectional transformer model, addressing the scarcity of high-quality annotated data and the challenge of model portability. While de-identification research in English clinical narrative texts [12–14] is extensive, the work on non-English clinical notes is limited. Grouin et al. [15] (2014). conducted a study on French clinical documents using the MEDINA suite to compare the efficacy of rule-based and CRF systems. Jian et al. [12] (2017) proposed a cascaded solution combining rule-based and machine learning methods to identify PHI from Chinese clinical notes. Wang et al. [16] (2022) utilized a variant of BERT as a feature extractor and the CRF model as a predictor, merging them to de-identify PHI in Chinese medical electronic health records. To our knowledge, the only existing study on de-identifying Korean medical notes was conducted by Shin et al. [17] (2015), who developed a rule-based approach with database matching and 15 regular expressions. However, the only a few types of PHI are covered by the developed regular expressions, some of which cannot even be applied to notes from other institutes. This lack of reliable methods has hampered the use of medical texts in clinical research.

## Objectives

The primary purpose of this study was to de-identify the radiology reports from Seoul National University Bundang Hospial (SNUBH), a tertiary university hospital located in a metropolitan area in South Korea, which are written in a mixture of Korean and English. We developed two different de-identification strategies and evaluated them to determine their effectiveness. The first was the rule-based approach, in which extensive regular expressions were developed to cover six PHI categories using 1,112 notes annotated by domain experts. In total, 51 regular expressions were developed. In the second approach, the developed regular expressions were used as labelers to pseudo-label a large number of notes and fine-tune a pre-trained Korean BERT model with them. The second approach can be considered a semi-supervised approach, as auto-labelling is used to increase the training data. The existing Korean named-entity-recognition (NER) model, KoBERT-NER, was used as the baseline model for training. By building regular expressions and evaluating strategies to combine them with machine learning, our research facilitates the secondary use of de-identified clinical documents.

## Methods

**Fig. 1:**
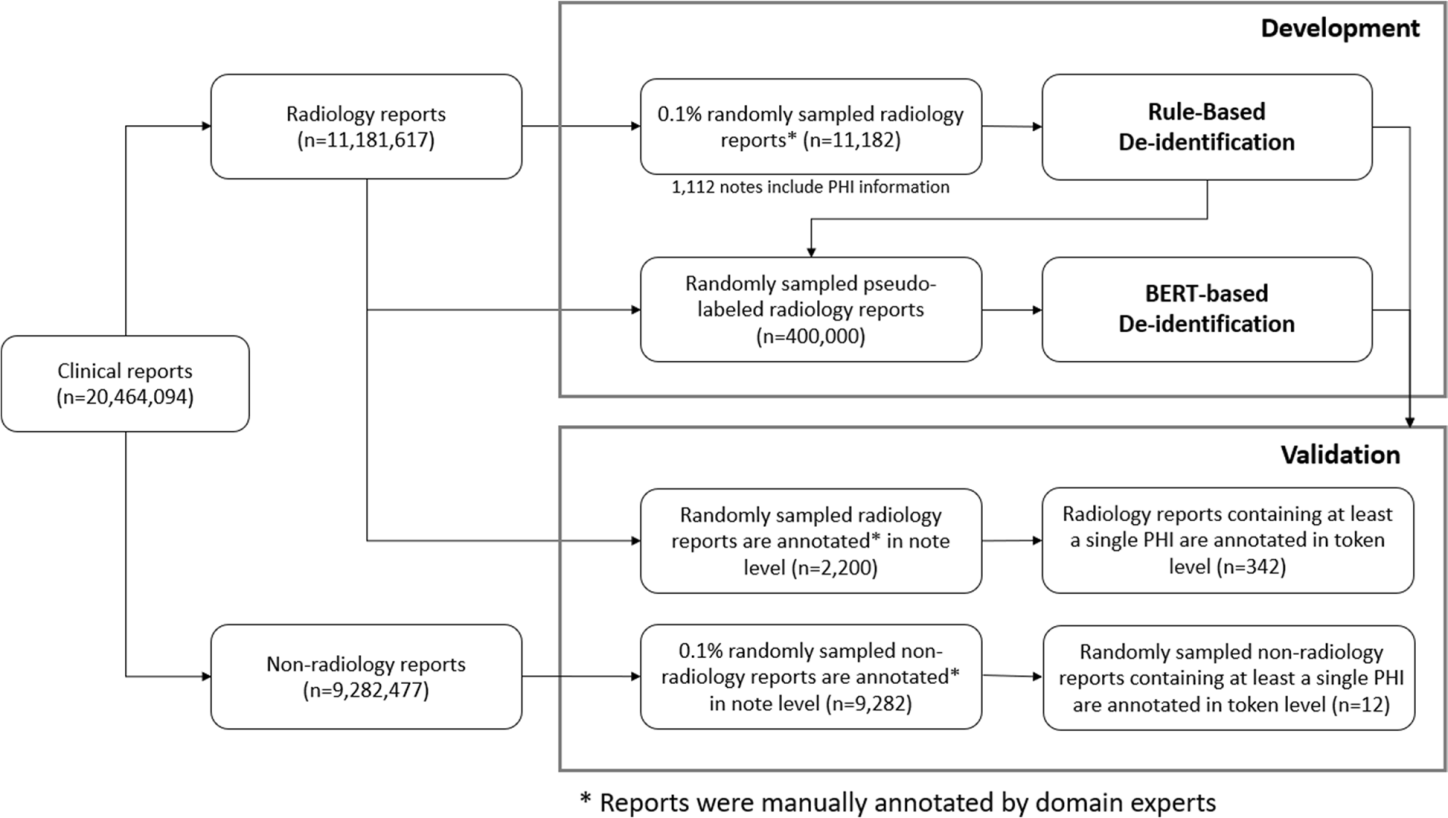
Process of de-identification using regex and BERT

Figure 1 illustrates the research process. Of the 11,181,617 radiology reports in the SNUBH database, we annotated 0.1% of the randomly selected documents (11,182) to ensure a representative sample of the overall dataset. This random sampling was designed to avoid any selection bias and reflects the broader dataset, both in structure and the distribution of PHI-containing notes. Among the annotated documents, 1,112 contained PHI. Based on the annotated documents, we built 51 regular expression rules, as described in the following sections. The constructed regular expression rules were utilized as pseudo-labelers to generate training data to fine-tune the BERT-based models. For the validation data, we prepared 342 notes and labeled them at the token level. The performance of each method on the notes of other departments was separately measured on 12 functional test reports from various departments (Department of Neurology, Department of Rehabilitation Medicine, and Endoscopy Center). The steps are described in detail in the following subsections.

### Data preparation

All notes for constructing regular expression rules were provided by the Department of Radiology at SNUBH. Manually annotated notes were selected as the ground truth. The PHI categories were DATE, NAME (both medical staff and patients), HOSPITAL, REGION, NUMBER (extension number of SNUBH, patient number), and other miscellaneous categories (nationality, sex, age), referring to the request of SNUBH.

As described in Table 1, approximately 10% (1,112) of the total 11,182 notes contain PHI words. The most frequent PHI was Date (94%), and the following were names of medical staff (5%) and names of patients (4.5%).

**Table 1:** The number of PHI words of documents used to build regular expression rules

Date	Name (medical staff)	Name (patients)	Hospital	Region	Numb	ETC	non-PHI	Total
1045	56	50	47	7	3	5	10,070	11,182

### Regular expression

**Fig. 2:**
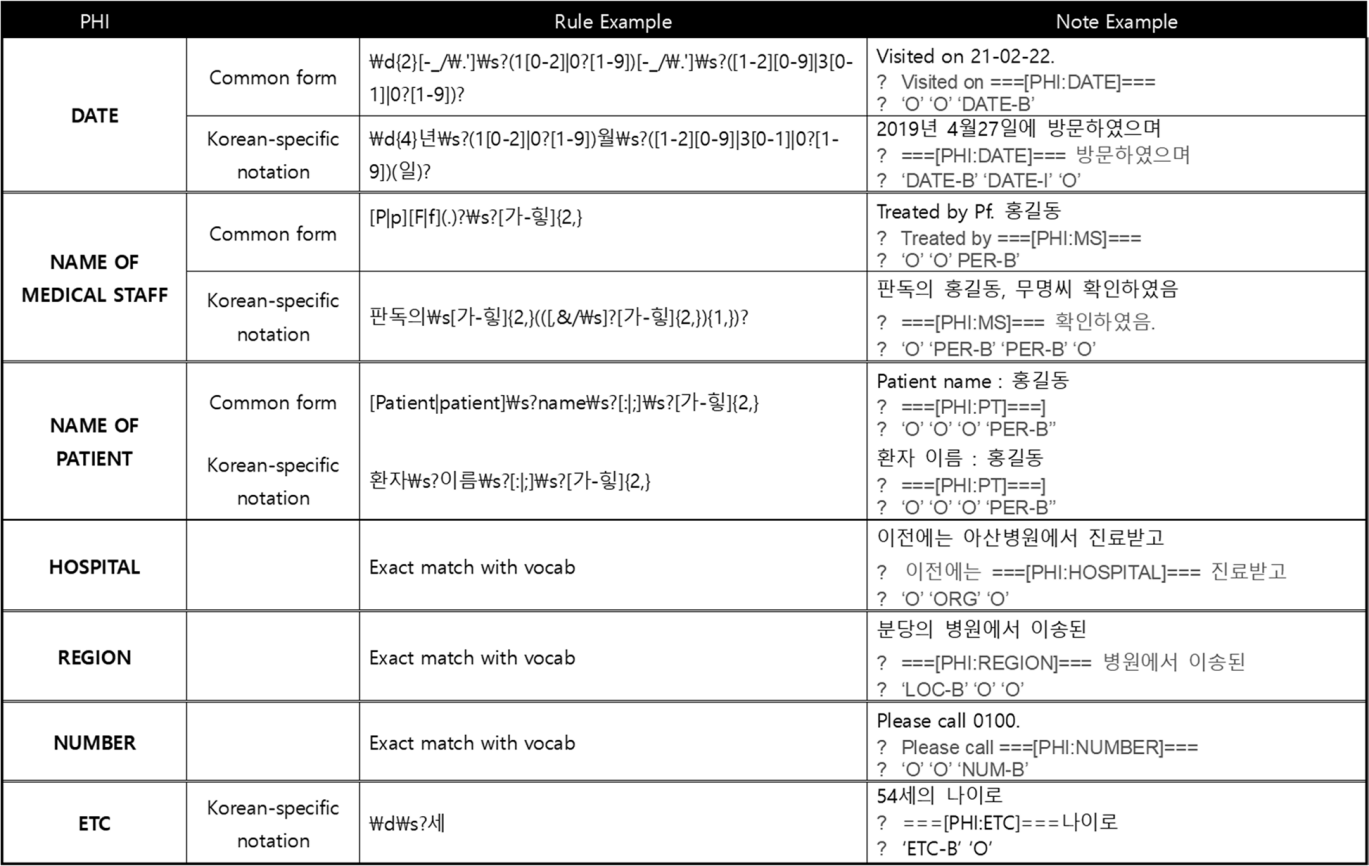
Examples of the regular expression rules and the results of notes

Regular expression rules were constructed after identifying repeating patterns from clinical notes. Figure 2 shows examples of regular expressions. Detailed information for each category is described below.

**Fig. 3:**
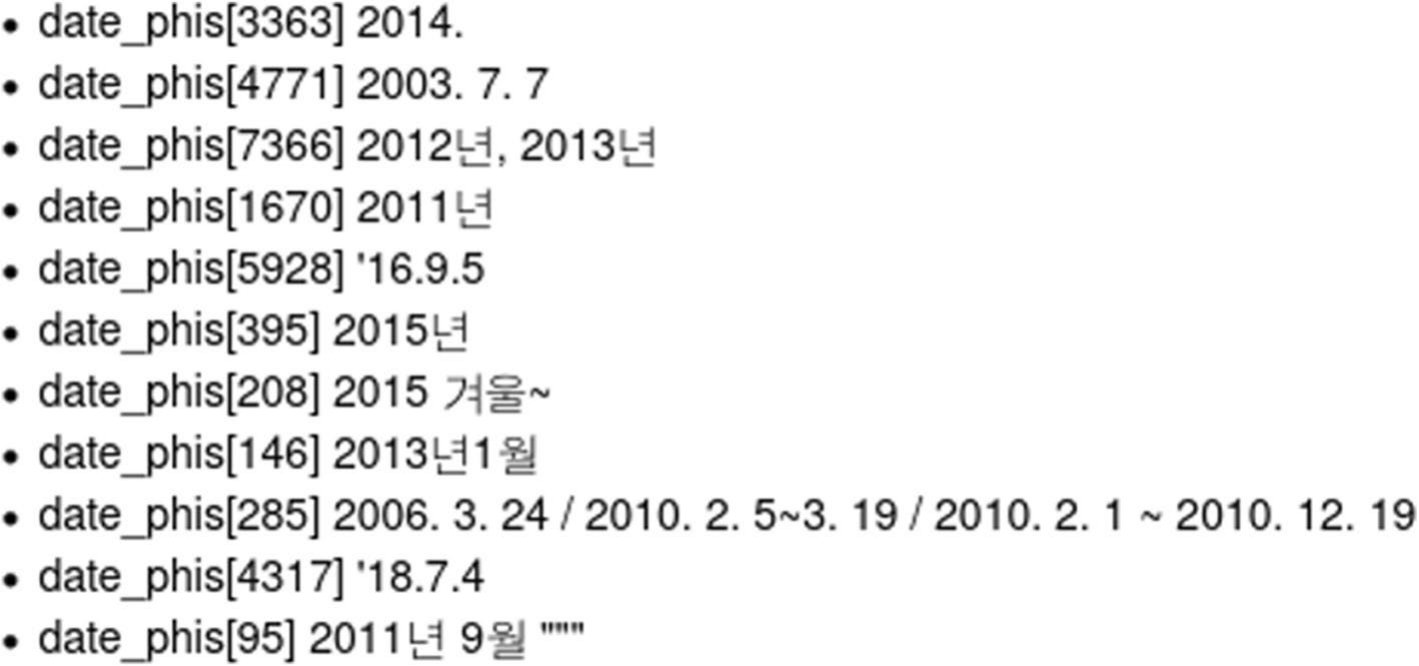
Examples of DATE notations written by several physicians

DATE If an expression has a year, month, and date with corresponding numbers, it is classified as a DATE. However, as shown in Fig. 3, a mix of English and Korean results in different types of DATE notation. Owing to the different writing styles of each physician and the use of bilingual expressions, 19 additional rules were added to detect patterns such as Korean-specific date notation (년(year), 월(month), 일 (day)), a period(.) and quotation marks (“).

NAME OF MEDICAL STAFF, NAME OF PATIENT eight regular expression rules for NAME of MEDICAL STAFF, and three rules for NAME OF PATIENTS were developed. The NAME OF THE MEDICAL STAFF has specific contexts. (1) A person who takes action such as “NAME 확인함, " “confirmed by NAME, " “from NAME. " (2) A person whose job is marked as “의료진 (medical staff) “, “판독의 (reading radiologist)”, or “교수 (professor)”. If these patterns appeared in a note, they were masked as the name of the medical staff. NAME OF PATIENTS was described as “환자이름 (patient name): NAME”. To separate the medical staff and patients, all patients were mentioned once with these rules in all notes. In addition, certain non-PHI words were utilized as identifiers because they often appear with PHI words. For example, in Fig. 2, “환자이름 (patient name)” and “판독의” are non-PHI words but were included in our regular expression rules to capture PHI (name of the patient and medical staff).

ORGANIZATION Thirteen rules were developed for the ORGANIZATION category. Even the same organization is referred to differently. For example, “서울대병원 (Seoul National University Hospital)”, “연건”, and “본원” refer to the same hospital. Similarly, “고려대학교병원 (Korea University Hospital)”, “고려대병원”, “고대병원”, “고대안암” and “안암” refer to the same institution. To process the abbreviations used by medical staff, vocabulary and regular expressions were built. By manually selecting a list of well-known hospitals in Korea, the following rules were established: (1) Word with “{병원}(hospital)” (2) Word with “{대학교}(university)” (3) Vocabulary to catch patterns. To identify non-major hospitals, which were not included in the vocabulary, the following rules such as ‘(병원|의원)(hospital|clinic)’, ‘[가-힣]피부과([a-z][ dermatology]). ’ were established Consequently, HOSPITAL has the second largest number of regular expressions after DATE, despite the small number of documents.

REGION, NUMBER, etc. The REGION category has only one rule. In many cases, LOCATION almost overlaps with ORGANIZATION because the physicians describe most of the regions when referring to HOSPITALS, e.g.“대구 (Daegu) local”, “김포우리병원 (Gimpo-Woori-Hospital)”, “강동성심병원 (Gangdong-SungsimHospital)”. Therefore, some specific city names, combined with well-known hospitals such as Bundang and Gangdong, were identified as ORGANIZATION. Four rules were developed for the NUMBER category, which comprises the extension number of SNUBH and patient number. However, because the hospital uses a combination of four digits as an extension number, distinguish them from the year is challenging. Therefore, the complete set of extension numbers for SNUBH was collected and a vocabulary set was constructed to match them manually. “전화번호 (telephone number)”, “Tel.“, “T.“, and “환자번호 (patient number)” were used as identifiers to detect NUMBER. Only five notes had the ETC category, which includes the patient’s age, sex, and nationality. The patterns corresponding to each note were added using four regular expressions.

### Machine-learning and pseudo-labeling

KoBERT [18] is a BERT-based Korean-language model developed by SKTBrain. KoBERT has 92 M parameters pre-trained with 5 M sentences from public Wikipedia and News. KoBERT-NER [19] is a named-entity recognition (NER) KoBERT model trained with the Naver NLP Challenge 2018 dataset, which has 81,000 training and 9,000 validation sets [20]. The types of labels used to train KoBERT-NER are similar to those of the PHI categories. The “Base” training level refers to a model trained using only the entity name extraction of the Naver NLP challenge 2018. This dataset contains newspaper articles and automatically generated sentences, which are more structured than other narrative texts but do not include clinical notes. Five out of the six PHI categories (DATE, PERSON, ORGANIZATION, LOCATION, and NUMBER) were included in the Naver NLP dataset; ETC (age, sex, and nationality) was not included.

**Fig. 4:**
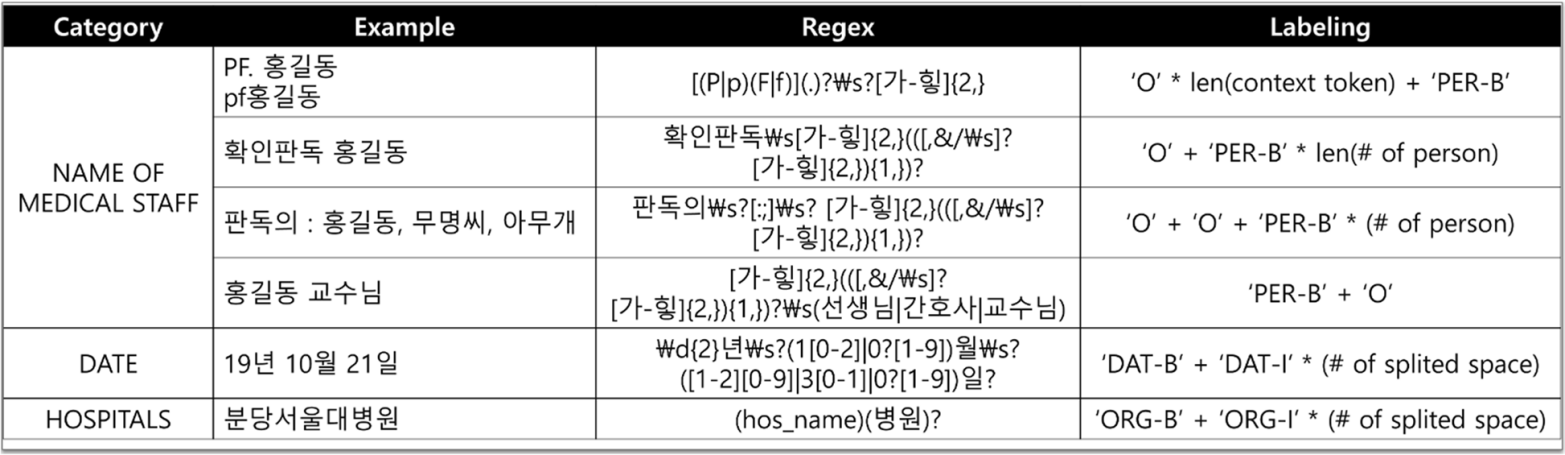
Examples of the pseudo-labeling formula of some categories

Pseudo-labels were obtained from the regular expressions we developed, as shown in Fig. 4 When labeling, not all the original KoBERT-NER labels were used. Instead, parts of the output were integrated into the predefined label for convenience. The NAME OF MEDICAL STAFF and NAME OF PATIENT were labeled as [PER], DATE as [DAT], HOSPITAL as [ORG], REGION as [LOC], NUMBER as [NUM], and ETC as newly defined [ETC]. In the original KoBERT-NER, the context of each label is slightly different from that in a medical record. For example, [DAT] covers expressions such as “today” or “the end of 2019” in the original version of KoBERT-NER. However, specific dates such as “2019-02-04” and “2020년 3월 7일” were used more frequently in the clinical notes than in the typical narrative notes. Similarly, [NUM] dealt with all numbers, but the model was only trained on four digits of the phone numbers. Although it does not precisely match the relationship between tokens and labels used in the original KoBERT-NER, the work was performed in as similar a context as possible.

Furthermore, from 400,000radiology reports, 87,015 PHI words were pseudo-labeled using the rule-based approach. These PHI words were distributed across 48,684 notes, each containing at least one PHI category. To compare the performance of models using different data sizes, 8,000, 16,000, and 32,000 notes (only PHI) were selected from 48,684 notes, which were classified as “Small”, “Medium”, and “Large 1”, respectively. The Large 2 dataset is a manually corrected version of Large 1 dataset. In Large 2, 295 false positives were removed from Large 1, resulting in a more accurate dataset. Additionally, 179 notes from non-radiology reports were added to Large 2 to improve the model’s performance on non-radiology notes, addressing the limitations of a radiology-only training set. In total, Large 1 contained 32,000 radiology notes, while Large 2 consisted of 31,705 corrected radiology notes and 179 additional non-radiology notes, leading to a more diverse training set for better generalization. The numbers of PHI words at each level are presented in Table 2.

**Table 2:** The number of PHI words for training data

Training level	DAT	PER	ORG	NUM	LOC	ETC
**Small (8**,**000)**	12,618	721	847	17	8	15
**Medium (16**,**000)**	25,425	1475	1765	39	15	25
**Large1 (32**,**000)**	50,907	2909	3522	190	79	143
**Large2 (31**,**884)**	51,158	2969	1957	215	83	272

In addition to KoBERT, we fine-tuned SciBERT [21], which is a BERT-based model trained on large-scale scientific articles. SciBERT has achieved state-of-the-art results for several NLP tasks in the scientific domain. In particular, Tai et al. [22] (2020). showed that the SciBERT-based NER model achieved the highest F1-score for biomedical NER performance. For comparison with KoBERT, we fine-tuned SciBERT using different levels of training data in the same manner as that for KoBERT.

## Results

To evaluate the performances of the methods, we manually annotated an additional 2,200 notes that were not included in the training set. Of the 2,200 notes, 342 contained at least one PHI word and were used as the test set. A total of 615 PHI words were identified from 342 notes (Table 3). The performance of non-radiology notes was tested separately on 12 annotated functional test reports from various departments, which together contained 68 PHI words. The samples of de-identification result are illustrated in Supplementary Fig. 2.1 and Supplementary Fig. 2.2.

**Table 3:** The number of PHI words in the test notes

Source	DAT	PER	ORG	NUM	LOC	ETC	Total
**Department of Radiology**	537	30	27	7	9	5	615
**Non-Radiology**	11	27	4	6	1	19	68

### Rule-based approach

Regular expressions performed well on the test set with a structure similar to that of the training set. However, several unmatched texts were detected when the notes contained text formats outside the scope of regular expressions. For example, (1) “1.2–1.7” could indicate either the period of the DATE or the medical term. However, being primarily composed of numbers, distinguishing between the two is difficult. (2) “상기확인함 by 홍길동” means medical staff “홍길동” was confirmed in one note. However, the regular expression catches both “NAME 확인함” and “by NAME” simultaneously; thus, “상기” was tagged as NAME although it should have been tagged as “O”. (3) “CMC” could be the “CATHOLIC MEDICAL CENTER,” which is classified as PHI under the ORG category, or it could refer to a joint area, which is not PHI. Unfortunately, our rule-based system does not distinguish between these contexts and consistently tags “CMC” as PHI.

Table 4 presents the performance of the rule-based method. The proposed regular expression achieved a precision, recall, and F1 score of 97.2%, 93.7%, and 96.2%, respectively on the test notes from the Department of Radiology. Of the 537 words classified as DAT, only one non-PHI and six PHI were incorrectly unmasked. Of the 30 identifiers in PER, the regular expression method masked 27 words correctly, but masked two non-PHI words. Since the slash (“ /“) was included to mark the initial of physicians, i.e., " /jmk/“ and “///skd/“, which is a general notation, false positives also increased. ORG has a similar problem. Among the 27 PHI words, 22 were correctly identified, but 4 were incorrectly masked because certain common Korean words have the same spelling as the hospital name. Although most rules marked PHI words correctly, they have distinct limitations owing to their inability to understand semantic properties. In addition, they are vulnerable to typos and notations lie outside their scope. For example, regular expressions designed for masking organization names such as " 고려대병원 (Korea University Hospital” and " 중앙대학교 (Chung-Ang University)” inadvertently masked unrelated text due to lexical ambiguities. The term “고려 (considering)” was incorrectly labeled as an organization (“ORG”) in the sentence " *이 lesion의 orientation을 고려해 볼 때 enlarged GB로 생각됨*”. Similarly, a rule targeting “중앙대학교 (Chung-Ang University)” mistakenly masked the term “중앙값 (median)”, highlighting the challenge posed by homonyms in Korean text.

**Table 4:** Result of regular expression

Test data	Department of Radiology			Other Department		
	Precision	`Recall	F1 Score	Precision	Recall	F1 Score
**DAT**	0.99	0.99	0.99	0.85	1.00	0.92
**PER**	0.93	0.83	0.88	1.00	0.45	0.62
**ORG**	0.79	0.82	0.80	1.00	1.00	0.91
**NUM**	1.00	0.71	0.83	1.00	0.83	0.91
**ETC**	1.00	1.00	1.00	1.00	0.95	0.97
**Total**	0.97	0.93	0.96	0.95	0.71	0.81

### Machine-learning approach

As shown in Table 5, the performance of the off-the-shelf KoBERT-NER (Base KoBERT-NER) is notably low, which indicates the need to use medical text for fine-tuning. As expected, the overall performance increases with the number of notes used for fine-tuning. Large 1 level trained KoBERT-NER achieves a precision, recall, and F1 score of 96.5%, 97.6%, and 97.1%, respectively, thereby outperforming the rule-based methods. Most importantly, recall, which is more important than precision in PHI de-identification, was particularly improved. Especially in PER, which requires contextual interpretation, the recall of Large 1 was 0.93, which was substantially higher than that (0.83) of the rule-based approach. The recall of ORG also substantially improved (0.82 vs. 0.96). Interestingly, the machine learning approach does not improve the performance in the NUM category. This unexpected result can be attributed to the small number of corresponding PHI words in the training dataset (Table 2). Large 2 level trained KoBERT-NER shows that removing the falsely detected words in the training data can significantly improve the model’s precision.

**Table 5:** Results of KoBERT-NER by training level

Training level	Base KoBERT-NER (0)		Small (8,000)		Medium (16,000)		Large1 (32,000)		Large2 (31,884)	
	Precision	Recall	Precision	Recall	Precision	Recall	Precision	Recall	Precision	Recall
**DAT**	0.49	0.43	0.87	0.74	0.81	0.79	0.99	0.98	0.98	0.99
**PER**	0.46	0.40	0.95	0.70	0.83	0.81	0.89	0.93	0.96	0.93
**ORG**	0.02	0.47	0.31	0.40	0.33	0.80	0.69	0.96	0.84	0.96
**NUM**	0.02	0.77	0.75	0.38	1.00	0.43	1.00	0.57	1.00	0.57
**LOC**	0.50	0.11	0.40	0.22	0.67	0.44	0.81	0.78	0.86	0.89
**ETC**	0.0	0.0	0.25	0.20	1.00	0.60	1.00	1.00	1.00	1.00
**Total**	0.46	0.43	0.83	0.71	0.79	0.7	0.96	0.97	0.98	0.98

Precision and Recall of each training level. Number in parentheses for each level indicates the number of notes in a train dataset for fine-tuning. As the number of trained notes increased, the accuracy of all categories increased

Notes from other departments not only contain different forms and sentence structures but also include new vocabulary. Being fine-tuned solely on notes from the department of radiology, the Large 1 level model did not perform well on the notes of other departments because of the forms of the notes and PHI categories are different from those of radiology notes. For example, PHI, such as “환자번호 (the identification number of a patient)” would lower the accuracy because it is not included in radiology notes. Meanwhile, the Large 2 level model, which included only 179 notes from non-radiology data into the training set, showed significant improvements in performance,, as shown in Table 6.

**Table 6:** Results of KoBERT-NER on non-radiology notes

Training level	Large 1 (32,000)			Large 2 (31,884)		
	Precision	Recall	F1 Score	Precision	Recall	F1 Score
**DAT**	0.52	1.00	0.69	0.52	1.00	0.69
**PER**	0.93	0.55	0.68	1.00	0.93	0.96
**ORG**	0.57	1.00	0.73	1.00	1.00	1.00
**NUM**	0.0	0.0	0.0	1.00	1.00	1.00
**LOC**	1.00	1.00	1.00	1.00	1.00	1.00
**ETC**	1.00	0.37	0.63	0.95	0.95	0.95
**Total**	0.78	0.56	0.62	0.91	0.96	0.93

#### Different base pre-trained model

We fine-tuned SciBERT using “Base” level and “Large 1” level. The results, as shown in Table 7, were measured on test notes from the Department of Radiology. The results from “Base” level show that both KoBERT and SciBERT fail to properly detect PHI words without fine-tuning using domain-specific data. Furthermore, in the “Large 1” level, SciBERT-NER achieved better precision while KoBERT-NER achieved better recall.

**Table 7:** Result of KoBERT-NER and SciBERT-NER

Training level	Base KoBERT-NER (0)		SciBERT-Base (0)		KoBERT-Large (32,000)		SciBERT-Large (32,000)	
	Precision	Recall	Precision	Recall	Precision	Recall	Precision	Recall
**DAT**	0.49	0.43	0.34	0.05	0.99	0.98	0.98	**1.00**
**PER**	0.46	0.40	1.00	0.40	0.89	0.93	1.00	0.79
**ORG**	0.02	0.47	0.05	0.43	0.69	0.96	0.79	0.87
**NUM**	0.02	0.77	0.01	0.54	1.00	0.57	1.00	0.57
**LOC**	0.50	0.11	0.0	0.0	0.81	0.78	1.00	0.67
**ETC**	0.0	0.0	0.0	0.0	1.00	1.00	1.00	0.54
**Total**	0.46	0.43	0.35	0.09	0.96	0.92	0.97	0.78

## Discussion

In this study, we adopted a rule-based approach and a pseudo-label-trained machine-learning approach to deidentify PHI with a limited amount of annotated data. Although regular expression rules require labor-intensive work to construct, they perform relatively well in the corresponding domain because the clinical notes are relatively structured compared to typical narrative texts. The 51 regular expressions developed in this study achieved a precision, recall, and F1 score of 97.2%, 93.7%, and 96.2%. However, generalizing and applying the rule-based approach to other department texts is difficult as they were solely constructed based on patterns in radiology reports from a specific hospital in Korea.

In the machine learning approach, off-the-shelf pre-trained NER models cannot be directly utilized in PHI de-identification because they were built based on different forms of notes. In our evaluation, Base KoBERT-NER and SciBERT-Base performed poorly. To improve performance, we fine-tuned KoBERT-NER using pseudo-labeled clinical notes generated by the rule-based method. Compared to the approach based solely on the rule, this approach significantly improved the performance, especially the recall of the PER and ORG. In short, the machine learning approach can utilize contextual information, which improves the performance.

Our experiments demonstrated that generalizing these methods and applying them to other departments or hospitals is possible, as evidenced by the fact that the performance of KoBERT-NER on the narrative notes in a specific domain increases as the amount of training data increases. In particular, we showed that adding a small number of notes from other departments to training data remarkably enhances a model’s capability to detect PHI patterns in other departments’ notes.

Although tokenization of medical terminologies may not be a critical issue for the PHI de-identification task, incorrect tokenization can debase the language understanding of a model. In our experiments, neither KoBERT nor SciBERT properly tokenized the majority of domain-specific medical terminologies. Therefore, further research should be conducted using a different base model that is pretrained with domain-specific corpora. Finally, we assume that the performance can be further improved if researchers add more identifiers to cover the outside scope of the current work and train a model with an extended version of pseudo-labeled notes.

We acknowledge that our evaluation was constrained by the limited size and diversity of the test set. A more comprehensive assessment using a larger external test set from different institutions would enhance our understanding of the models’ performance and generalizability. Unfortunately, data availability and collaboration constraints made this unfeasible within the scope of this study, but it remains a key direction for future research.

## Conclusion

In this study, we de-identified PHI from clinical notes using rule-based and machine learning methods. First, we constructed 51 regular expressions to cover 6 PHI categories using radiology reports from SNUBH. Subsequently, we used the developed regular expressions as a labeler to pseudo-label a large number of notes and fine-tuned KoBERT and SciBERT. By evaluating the performance of models that were fine-tuned in different settings, we confirmed that the performance of the models on the notes in a specific domain increases as the number of training data increased. Additionally, we significantly improved the performance of the model on non-radiology notes by adding a small number of non-radiology reports to the training data. This finding shows that generalizing our method and applying them to other departments and hospitals is possible even though the de-identification model is trained on limited training data. However, we acknowledge that our approach has limitations due to the possibility of the model learning inappropriate features from the pseudo-labeled notes generated by the rule-based method because regular expressions cannot distinguish homonyms and are susceptible to typos. In our study, this problem resulted in relatively low precision of the model on some categories. While we were able to address this issue by making manual corrections on pseudo-labeled notes, it is necessary to conduct further research to find a solution that does not require human intervention.

## Supplementary Information

Supplementary Material 1.

Supplementary Material 2.

Supplementary Material 3.

## Data Availability

The data used in this study cannot be shared owing to a policy of the institutional review board of SNUBH. The corresponding author (yoosoo0@snubh.org) can be contacted regarding a request on data and materials. Python code and regular expressions used in this study, along with an example clinical note for reproduction purposes, are available in https://github.com/leelabsg/SNUBH_deid.

## Abbreviations

BERT: Bidirectional Encoder Representations from Transformers
EHR: Electronic Health Records
KoBERT: Korean Bidirectional Encoder Representations from Transformers
NER: Named Entity Recognition
NLP: Natural Language Processing
OMOP: Observational Medical Outcomes Partnership
PHI: Protected Health Information
