# Supplementary material for: De-identification of clinical notes with pseudo-labeling using regular expression rules and pre-trained BERT
Source: BMC Med Inform Decis Mak. 2025 Feb 17;25:82. doi: 10.1186/s12911-025-02913-z (PMC11831849; doi:10.1186/s12911-025-02913-z)
Supplement: Supplementary file 1 — Supplementary Material 1. [file 12911_2025_2913_MOESM1_ESM.pdf]

Title: De-Identification of Clinical Notes with Pseudo-labeling using Regular Expression Rules and Pre-trained BERT

Jiyong An, Jiyun Kim, Leonard Sunwoo, Hyunyoung Baek, Sooyoung Yoo, Seunggeun Lee

Supplementary 1. Details of Bert Fine-tuning

BERT (Bidirectional Encoder Representations from Transformers) is a transformer-based model distinguished by its bidirectional architecture. The architecture consists of multiple layers of Transformer units, each equipped with multi-head Attention mechanisms that facilitate the capture of diverse semantic relationships from various perspectives. The initial pre-training of BERT involves two primary strategies, which are the Masked Language Model (MLM) and Next Sentence Prediction (NSP). In the MLM approach, a certain portion of the tokens within a sentence are randomly replaced with a [MASK] token, compelling the model to predict the original tokens based on the surrounding unmasked context. Conversely, NSP trains the model to determine whether one sentence logically follows another, enhancing its capability to understand textual relationships.

Following its pre-training, BERT undergoes a fine-tuning process tailored to specific NLP tasks, involving modifications to the model’s output layers to suit particular applications. In this step, the weights from the pre-trained model are fine-tuned using the gradient descent algorithm, focusing on minimizing the cross-entropy loss between the predicted and actual labels. For token-level tasks such as Named Entity Recognition (NER), a token classification layer is integrated. This layer employs a softmax function to predict a label for each token in the sequence, corresponding to categories of named entities like person names, organizations, or locations.

In this study, we employed the KoBERT-NER model, a variant of KoBERT specifically fine-tuned for NER tasks using the Naver NLP Challenge 2018 dataset. Hyperparameters included a batch size of 32, a learning rate of 5e-5, and 20 training epochs. We utilized the Adam optimizer with an epsilon of 1e-8. For full transparency and to facilitate future research and replication of our results, the complete training code, including scripts for model setup, data processing, and evaluation, is publicly available on our GitHub repository at https://github.com/leelabsg/SNUBH_deid.git.
