# Supplementary material for: De-identification of clinical notes with pseudo-labeling using regular expression rules and pre-trained BERT
Source: BMC Med Inform Decis Mak. 2025 Feb 17;25:82. doi: 10.1186/s12911-025-02913-z (PMC11831849; doi:10.1186/s12911-025-02913-z)

Title: De-Identification of Clinical Notes with Pseudo-labeling using Regular Expression Rules and Pre-trained BERT

Jiyong An, Jiyun Kim, Leonard Sunwoo, Hyunyoung Baek, Sooyoung Yoo, Seunggeun Lee

Supplementary Figure 2.1 Results containing Single label

Actual PHI informations have been arbitrarily replaced.

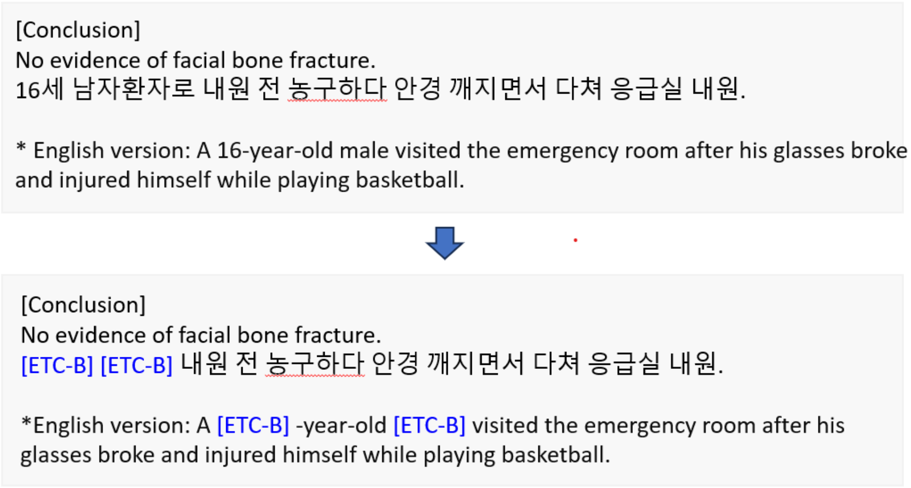

Supplement: Supplementary file 2 — Supplementary Material 2. [file 12911_2025_2913_MOESM2_ESM.pdf]
