# Supplementary material for: De-identification of clinical notes with pseudo-labeling using regular expression rules and pre-trained BERT
Source: BMC Med Inform Decis Mak. 2025 Feb 17;25:82. doi: 10.1186/s12911-025-02913-z (PMC11831849; doi:10.1186/s12911-025-02913-z)
Supplement: Supplementary file 3 — Supplementary Material 3. [file 12911_2025_2913_MOESM3_ESM.pdf]

Title: De-Identification of Clinical Notes with Pseudo-labeling using Regular Expression Rules and Pre-trained BERT

Jiyong An, Jiyun Kim, Leonard Sunwoo, Hyunyoung Baek, Sooyoung Yoo, Seunggeun Lee

Supplementary Figure 2.2 Results containing Multiple labels

Actual PHI informations have been arbitrarily replaced.

[Finding]
C.I> #pituitary adenoma -2020.3 TSA and tumor removal ((A병원) – 2020.5.29 TSA and tumor removal (revision Op) (Pf 홍길동)
Comparison: 2020-05-13 CT
Immediate postop.study

[Finding]
C.I> #pituitary adenoma -2020.3 TSA and tumor removal (([ORG-B]) – 2020. 5.29 TSA and tumor removal (revision Op) (Pf [PER-B])
Comparison: [DAT-B] CT
Immediate postop.study
